# Why is Cognitive Enhancement Deemed Unacceptable? The Role of Fairness, Deservingness, and Hollow Achievements

**DOI:** 10.3389/fpsyg.2016.00232

**Published:** 2016-02-19

**Authors:** Nadira S. Faber, Julian Savulescu, Thomas Douglas

**Affiliations:** ^1^Department of Experimental Psychology, University of OxfordOxford, UK; ^2^Oxford Martin School, University of OxfordOxford, UK; ^3^Uehiro Centre for Practical Ethics, Faculty of Philosophy, University of OxfordOxford, UK; ^4^Brasenose College, University of OxfordOxford, UK

**Keywords:** cognitive enhancement, neuroenhancement, brain doping, drugs, fairness, cheating, moral judgments

## Abstract

We ask why pharmacological cognitive enhancement (PCE) is generally deemed morally unacceptable by lay people. Our approach to this question has two core elements. First, we employ an interdisciplinary perspective, using philosophical rationales as base for generating psychological models. Second, by testing these models we investigate how different normative judgments on PCE are related to each other. Based on an analysis of the relevant philosophical literature, we derive two psychological models that can potentially explain the judgment that PCE is unacceptable: the “Unfairness-Undeservingness Model” and the “Hollowness-Undeservingness Model.” The Unfairness-Undeservingness Model holds that people judge PCE to be unacceptable because they take it to produce unfairness and to undermine the degree to which PCE-users deserve reward. The Hollowness-Undeservingness Model assumes that people judge PCE to be unacceptable because they find achievements realized while using PCE hollow and undeserved. We empirically test both models against each other using a regression-based approach. When trying to predict judgments regarding the unacceptability of PCE using judgments regarding unfairness, hollowness, and undeservingness, we found that unfairness judgments were the only significant predictor of the perceived unacceptability of PCE, explaining about 36% of variance. As neither hollowness nor undeservingness had explanatory power above and beyond unfairness, the Unfairness-Undeservingness Model proved superior to the Hollowness-Undeservingness Model. This finding also has implications for the Unfairness-Undeservingness Model itself: either a more parsimonious single-factor “Fairness Model” should replace the Unfairness-Undeservingness-Model or fairness fully mediates the relationship between undeservingness and unacceptability. Both explanations imply that participants deemed PCE unacceptable because they judged it to be unfair. We conclude that concerns about unfairness play a crucial role in the subjective unacceptability of PCE and discuss the implications of our approach for the further investigation of the psychology of PCE.

## Introduction

### Pharmacological Cognitive Enhancement and its Perceived Unacceptability

There are a number of means to enhance cognitive capacities beyond what is usually seen as compensation for an impairment. Nutrition and physical exercise improve cognitive functioning in healthy people across different domains (e.g., Dresler et al., 2013), whilst commonplace stimulants such as caffeine temporarily boost functions like alertness and concentration (e.g., Einöther and Giesbrecht, 2013). Use of these techniques is uncontroversial. Far more controversial is so-called “brain doping," that is the use of “pharmacological interventions that are intended to improve certain mental functions and that go beyond currently accepted medical indications" (Schermer et al., 2009, p. 77).

Such pharmacological cognitive enhancement (PCE) may be achieved through the use of psychostimulants like methylphenidate (e.g., Ritalin^®^) and wakefulness-promoting drugs like modafinil (e.g., Provigil^®^). Research has demonstrated that these substances can have performance-enhancing effects in healthy individuals, for example by improving memory or attention (for reviews, see Repantis et al., 2010; Battleday and Brem, 2015; Ilieva et al., 2015). However, current PCE cannot enhance performance to more than modest degrees at best, depending on individual baseline performance (Husain and Mehta, 2011; Caviola and Faber, 2015). Some societies have witnessed a rise in the use of PCE (Care Quality Commission, 2013). Prevalence studies and informal polls suggest that at least some members of different groups use pharmacological substances with the goal to enhance their performance, for instance researchers (Maher, 2008), surgeons (Franke et al., 2013), and, across a range of countries, students (e.g., Singh et al., 2014; Maier et al., 2015; Schelle et al., 2015).

Pharmacological cognitive enhancement not only receives significant media attention, but is also intensively researched in a range of academic disciplines. These disciplines share the aim of understanding PCE (and mind-altering technologies in general) better, and helping society to deal with the challenges posed by increasing PCE use (cf. Greely et al., 2008; Smith and Farah, 2011; Sahakian et al., 2015). PCE is a truly interdisciplinary research topic, on which different disciplines can – and probably ought to – collaborate (cf. Hildt and Franke, 2013; Maslen et al., 2015). Neuroscience and the medical sciences investigate the pharmacological effects and potential side-effects of such substances (e.g., Turner et al., 2003). The behavioral and social sciences deal with questions such as what drives individuals to take PCE (e.g., Wolff and Brand, 2013), how members of the general public perceive PCE (e.g., Sattler et al., 2013), and which social consequences these perceptions might entail for users (e.g., Faulmüller et al., 2013). Meanwhile researchers in philosophy and law examine the ethical and legal problems PCE use entails, weigh these against possible benefits, and in some cases derive recommandations for public policy (e.g., Maslen et al., 2014a) and legal regulation (e.g., Goold and Maslen, 2014). In doing so, they rely on empirical research, as both findings on the pharmacological effects of PCE (e.g., Maslen et al., 2014b), as well as the public perception of PCE (e.g., Forlini et al., 2013) are crucial inputs into ethical, legal and policy debates regarding PCE.

Empirical studies on how members of the general public perceive PCE have already uncovered a variety of concerns people have about PCE, for example regarding medical safety (e.g., Scheske and Schnall, 2012) and societal inequality (e.g., Fitz et al., 2013; for a review, see Schelle et al., 2014). The – although often implicit – goal of many of these studies is to better understand one consistent finding, namely that PCE is deemed *morally unacceptable* (cf. Schelle et al., 2014). The judgment that “PCE is morally unacceptable” – henceforth abbreviated as “***Unacceptability***” – is also found in media reports and in much of the normative debate. This article addresses the question: *why* do lay people endorse *Unacceptability*? That is, why do they judge PCE to be morally unacceptable?

### The Present Research: Combining Philosophical Rationales and Psychological Explanations

The primary innovation of our contribution is to employ an interdisciplinary perspective that combines normative philosophical and empirical psychological analyses. We propose that this combination provides a fruitful way to deepen understanding of why people generally judge PCE to be morally unacceptable. Philosophers who have explored moral responses to PCE have frequently, amongst other things, been interested in normative rationales, less in psychological explanations. That is, they have often not been asking why, as a matter of fact, people endorse a certain judgment, but why it might be *rational* to endorse it. Thus, no psychological conclusions can be straightforwardly drawn from philosophical work. However, we suggest that philosophical rationales can be useful in generating psychological hypotheses. As shown in the review by Schelle et al. (2014), lay attitudes on PCE tend to coincide with the attitudes of professional philosophers, suggesting that lay attitudes may have partly the same bases as professional philosophical attitudes. In other words, both philosophical rationalizations and lay attitudes might in part be expressions of a common rational thinking process, which philosophers make more explicit than lay people. (It is important to note, however, that intuitive lay judgments on PCE seem not always to be fully rational, Scheske and Schnall, 2012; cf. Caviola et al., 2014). Philosophical rationales for attitudes on PCE could thus be thought of as making explicit the psychological mechanisms that motivate acceptance of these attitudes in both philosophers and lay people, in so far as both groups form these attitudes rationally. Hence, we explore how philosophical rationales may aid psychology in identifying credible explanations for lay endorsement of *Unacceptability*.

We test the role of three judgments in explaining *Unacceptability:* (1) “PCE produces unfair outcomes,” henceforth “***Unfairness*,***”* (2) “achievements realized with the aid of PCE are “hollow achievements” in the sense that they lack (some of their usual) value,” henceforth “***Hollowness*,**” and (3) “users of PCE do not deserve their achievements or the material and non-material reward associated with them,” henceforth “***Undeservingness.***” Based on philosophical literature we generate two explanatory psychological models which are based on *Unfairness*, *Hollowness*, and *Undeservingness*, and test these against empirical data.

Philosophers have, implicitly or explicitly, endorsed or at least considered not only *Unacceptability*, but also *Unfairness*, *Hollowness*, and *Undeservingness*. More importantly, recent applied philosophical work on the ethics of cognitive enhancement has begun to explore the relationships between these views, and related work in theoretical philosophy could be deployed to further develop this understanding. Empirical work, in contrast, has consistently shown that lay people are concerned about unfairness induced by PCE use (cf. Schelle et al., 2014), but has not tested whether achievements realized with the help of PCE are seen as hollow or as undeserved or investigated the relationships between these views. Hence, it remains unclear precisely which, if any, of the judgments *Unfairness*, *Hollowness*, and *Undeservingness* contribute to lay endorsement of *Unacceptability*. For instance, is the perceived unacceptability of PCE explained by the judgment that it produces unfair outcomes, the judgment that users of cognitive enhancements do not deserve the reward they received, by both, or by neither? In addition, though it is possible that some or all of these judgments *jointly* explain support for *Unacceptability*, it is not clear (I) what relative contribution each judgment makes to this explanation; and (II) how, if at all, they interact. In this paper, we complement existing empirical research on the question of why lay people endorse *Unacceptability* by comparing three different factors (*Unfairness*, *Hollowness*, and *Undeservingness*) with regard to their relative strength in explaining the overall judgment of *Unacceptability*. We build on existing work by examining two judgments (*Hollowness* and *Undeservingness*) that have not previously been empirically investigated and by examining how the three judgments we consider interact with each other.

In sum, in this paper we combine philosophical rationales and psychological explanations to investigate why PCE is judged as morally unacceptable. We first outline two possible rationales for *Unacceptability*, drawing on both applied and theoretical philosophical work. We then offer two psychological models grounded on these rationales—the Unfairness-Undeservingness Model and the Hollowness-Undeservingness Model—and spell out our research questions regarding these models. Next, we describe our methods for testing these two models against empirical psychological data using a regression-based approach, before setting out the results of this testing. Finally, we discuss the implications of our findings for the psychology and philosophy of PCE.

## Philosophical Rationales

The lay judgment we ultimately wish to explain—*Unacceptability*—holds that PCE is morally unacceptable. This judgment has been endorsed by a number of philosophers, who have considered a wide range of rationales for it. Broadly speaking, these can be divided into three categories: rationales that focus on the *motives* for which PCE is pursued (e.g., Little, 1998; Sandel, 2007), rationales that focus on the *means* by which it is pursued (e.g., The President’s Council on Bioethics (U.S.), 2003; Sandel, 2007), and rationales that focus on the *consequences* of pursuing it (e.g., Fukuyama, 2002; Elliott, 2003). In this section, our aim is not to offer a comprehensive review of all these rationales—this would be too ambitious a task (for a review, see Douglas, 2013). Rather, we limit ourselves to outlining rationales that meet two conditions. First, they appeal to one or more of the judgments *Undeservingness*, *Hollowness*, and *Unfairness* outlined above. Second, they are consequence-based, rather than motive- or means- based rationales. Our reason for limiting our discussion to consequence-based rationales is that adherents of a wide range of moral theories can accept such rationales. Almost all moral theories allow that an act or practice can be morally unacceptable because it has, or can be expected to have, bad consequences. By contrast, it is controversial whether an act can be morally unacceptable purely because of the means that it involves or the motives that produced it.^1^

Two prominently discussed rationales meet our two conditions, namely what we call the “objection from fairness” and the “objection from hollow achievements.” In what follows, we set out our interpretations of these rationales.

### The Objection from Fairness

A number of authors have endorsed, or at least considered, the view that PCE (or enhancement more generally) may be morally unacceptable because it is unfair or, perhaps equivalently, constitutes a form of “cheating” (e.g., Fukuyama, 2002; The President’s Council on Bioethics (U.S.), 2003; Rose, 2006; Schermer, 2008). We call this the “objection from fairness.” One variant of this objection holds that PCE is *procedurally* unfair: that it involves unfair means. This is a means- rather than consequence-based rationale for *Unacceptability*, and as such we do not discuss it further. A second variant of the objection holds that PCE is *substantively* unfair: that it produces unfair outcomes, as *Unfairness* holds. This variant of the objection is consequence-based, and will be our focus.

Two questions should be asked regarding this variant of the objection from fairness. First, why think that *Unfairness* supports *Unacceptability*? Second, why accept *Unfairness* in the first place?

The answer to the first question is straightforward, though not normally made explicit in the literature on PCE: unfairness is bad, and as noted above, proponents of a range of moral theories can agree that, other things being equal, it is morally unacceptable to produce bad consequences. Why, precisely, unfairness is bad is controversial. Some hold that it is bad *in itself* (e.g., Broome, 1991). Others, would deny this and hold that fairness is only bad if and because it tends to produce further bad consequences, such as reduced individual wellbeing (e.g., Bentham, 1789; Sidgwick, 1893). However, despite this disagreement about *why* unfairness is bad, many agree *that* it is bad, or at least typically so.

The second question—why should we accept *Unfairness*—has caused greater controversy in the ethical debate regarding PCE. On the one hand, it seems “obvious” (The President’s Council on Bioethics (U.S.), 2003, p. 280) or at least “intuitive” (Schermer, 2008, p. 88) that some instances of enhancement, including PCE, produce unfairness. On the other hand, doubts can be raised about whether *all* enhancements, or all PCEs, do so (e.g., Savulescu, 2006; Douglas, 2007; Sandel, 2007; Schermer, 2008; Buchanan, 2011a,b; Santoni de Sio et al., in press). Hence, the scope of application of *Unfairness* is contested. There is also disagreement about how to rationalize *Unfairness*, that is about *why* enhancement produces unfairness when it does. One rationale holds that enhancement involves violating social rules or conventions, and it is unfair if individuals acquire reward through rule-violations (cf. Schermer, 2008). As has been noted, however, those who raise fairness-based concerns regarding enhancement frequently take these concerns to apply regardless whether the enhancement in question violates a rule (Schermer, 2008; Savulescu, 2009). For instance, in the context of debate over enhancement in sport, concerns about production of unfairness have often been presented as a *justification* for maintaining prohibitions on enhancement rather than merely a consequence of such prohibitions (e.g., Lenk, 2007; Corlett et al., 2013). Similarly, philosophers concerned about fairness in relation to PCE have not generally restricted their concerns to rule-violating PCE (Fukuyama, 2002; The President’s Council on Bioethics (U.S.), 2003). Hence, it seems appropriate to seek a more general rationale for *Unfairness*—one that will apply even in cases where PCE does not involve rule-violation. We suggest that *Undeservingness* might be able to furnish such a rationale (cf. also Schermer, 2008).

As defined above, *Undeservingness* is the judgment that PCE-users do not deserve their achievements or the material (e.g., money) and non-material (e.g., praise) reward associated with them. A number of authors in the debate on the ethics of enhancement have explicitly considered this view (e.g., Mehlman, 2004; Schermer, 2008; Forsberg, 2013), and it has been suggested (Douglas, 2014) that a similar view may be implicit in the work of others (Harris, 2012; Sparrow, 2014). Moreover, opponents of PCE frequently advance claims that can be understood to support *Undeservingness.* For instance, although disputed elsewhere (Douglas, 2014), it is often said that enhancement makes achievements “too easy” or is a way of avoiding effort (Cole-Turner, 2000; Kass, 2003). If true, this might support *Undeservingness*, since exerting effort to overcome difficulties is often thought to confer deservingness (Sadurski, 1985; Milne, 1986; Sorensen, 2010).

The relationship between *Undeservingness* and *Unfairness* has not been explored in detail in the applied philosophical literature on PCE (although cf. Mehlman, 2004; Schermer, 2008); however, it is plausible that the two judgments are normatively connected. One possibility is that *Unfairness* rationalizes *Undeservingness*—that is, because users of PCE are the beneficiaries of unfairness, they do not deserve their reward. Intuitively, people do not deserve unfairly acquired benefits. For instance, when an athlete breaks the rules of a sport and, as a result, wins a competition, we would conclude that she has won unfairly, and this may seem to support the view that she does not *deserve* the reward that come with the victory. This sort of case might seem to suggest that *Unfairness* is normatively more fundamental than *Undeservingness*.

However, theoretical work on the nature of fairness suggests that *Undeservingness* may be the more fundamental judgment: *Undeservingness* may be able to support *Unfairness*.^2^ Some prominent theoretical accounts of fairness can be interpreted as holding that fairness, or at least one component of fairness, requires that (material or non-material) reward are distributed across individuals in proportion to the relative degree to which those individuals deserve those reward (Broome, 1990; Feldman, 1995a; Kagan, 2012). In support of this conception of the relationship between fairness and deservingness, consider the following case: Two charity workers undertake humanitarian projects in a poverty-stricken area without any expectation of reward. Their projects are very different in difficulty and scope. One spends several years single-handedly building a hospital that will save thousands of lives over the coming decades. The other spends one afternoon writing letters to local politicians, with the effect that those politicians divert an additional $500 to the provision of affordable pain relief medications. This can be expected to slightly increase the quality of life of each of 100 migraine sufferers for around a week. Intuitively, the first charity worker is more deserving than the second, all else equal, but it would be difficult to rationalize this judgment regarding deservingness by invoking the concept of fairness. On the other hand, the judgment regarding deservingness does seem potentially capable of rationalizing a judgment regarding fairness. Suppose both charity works receive similar levels of praise for their efforts. Intuitively, this is unfair. The first charity working deserves more praise, and it seems unfair if he does not get it.

A similar line of reasoning suggests that *Unfairness* may be able to rationalize *Undeservingness*. Imagine a case in which two scientists, *A* and *B*, make similar and highly significant scientific discoveries. Suppose, however, that *A* made her discovery assisted by PCE which allowed her to work longer hours and more productively, whereas *B* made the discovery without any such pharmacological assistance. Although we do not ourselves endorse this view, according to *Undeservingness, A* does not deserve her achievement or the praise, academic success, and other reward that accompany it, perhaps because her enhancement allows her to avoid effort, or made her achievement “too easy.” On the other hand, it is plausible to assume that *B* does deserve her achievement and associated reward, at least to some extent. However, despite this difference in deservingness, it is likely that these two scientists will receive a similar size of reward for these achievements, at least if *A*’s PCE-use is secret (cf. Faulmüller et al., 2013). Thus, rewards are not distributed in proportion to deservingness, and this, on the present conception of fairness, is unfair. Hence, if (1) *Undeservingness* holds true, and 2) PCE users are rewarded to a similar degree as non-users who achieve similar things, then use of PCE may disrupt fairness.

### The Objection from Hollow Achievements

A second candidate rationale for *Unacceptability* invokes *Hollowness*—the claim that achievements realized with the aid of PCE are “hollow achievements” in the sense that they lack (some of their normal) value. This claim, or variants thereof, have been endorsed by a number of authors in the ethical debate on PCE, and enhancement more generally. Juengst (2000) raised the question whether achievements realized via enhancement might be “hollow accomplishments” (p. 39), and The President’s Council on Bioethics (U.S.) (2003) claimed that enhancements would undermine the “dignity” (p. 140) of human performance and perhaps render that performance “false” (p. 131), thereby highlighting two specific values (dignity and truth) that enhancements might threaten. In what follows, we focus on the question whether PCE might deprive human achievements of *some* degree of value without taking a stance on what particular kind of value that might be. Following Juengst’s terminology, we call this the “objection from hollowness.”

As with the objection from fairness, two questions should be asked regarding the objection from hollowness. First, why think that *Hollowness* supports *Unacceptability*? Second, why accept *Hollowness*?

On the first question, why *Hollowness* supports *Unacceptability*, little has been said. However, it is possible to construct a straightforward argument from *Hollowness* to *Unacceptability*. According to *Hollowness*, achievements realized with the aid of PCE lack (some of their normal) value, and this means that pursuit of enhancement has at least one bad consequence: it diminishes at least some forms of value that our achievements might otherwise have had.

More has been said on the second question: *why* accept *Hollowness*? That is, why judge achievements gained with the help of PCE to be hollow achievements? On one view, PCE use can devalue achievements because it corrupts the very purpose of the activity being pursued (e.g., Santoni de Sio et al., in press). In this regard, using an enhancement might – to take an often-cited example – be like completing a marathon with the aid of roller skates (Whitehouse et al., 1997). Some activities (including marathon running) fulfill their purpose only where pursued in a certain kind of way, and in some cases enhancement is incompatible with the required manner of execution. This may be because the activities in question only have value when they manifest a certain kind of human contribution, and the use of enhancement somehow negates the need for any such contribution (Savulescu, 2015). However, as many have noted, not all activities are such that their purpose is undermined when they are pursued with the aid of enhancements (e.g,. Douglas, 2007; Bostrom and Roache, 2008; Roache, 2008; Schermer, 2008; Goodman, 2010; Santoni de Sio et al., in press). Consider landing an airplane or performing a surgical operation. The purpose of these activities is to realize a certain outcome, and the realization of that outcome need not be threatened, and may even be aided, by the use of enhancements (cf. Santoni de Sio et al., 2014). Moreover, activities that would be rendered hollow by very extensive enhancements may not be rendered hollow by more modest ones. For instance, climbing Mount Everest with the aid of a jetpack might render it a hollow achievement, but it is far less clear that climbing with the aid of compressed oxygen, or regular morning coffees, does so. Hence, we think that the present argument cannot support the claim that, generally, achievements realized via PCE are hollow, as some have suggested (e.g., The President’s Council on Bioethics (U.S.), 2003). As with *Unfairness*, then, it is desirable to seek a more general rationale for *Hollowness.* And as with *Unfairness*, we suggest that it may be possible to provide such a rationale by using *Undeservingness.*

It is often thought that things that are normally valuable can lack this value when they are not deserved. For instance, pleasure is normally valuable—it normally makes the world a better place when a person experiences pleasure—but some argue that it lacks its normal value when it is not deserved (e.g., Brentano, 1969; Feldman, 1995b). Hence, on this view, pleasure is, other things being equal, less valuable when it is enjoyed by a mass-murderer than when it is enjoyed by an innocent person. Similar thoughts may apply to valuable achievements. It may be that, when achievements are underserved, they lack value. If so, and if PCE undermines deservingness, then achievements realized with the aid of PCE lack value—that is, *Hollowness* holds true.^3^

### The Unfairness-Undeservingness Model and the Hollowness-Undeservingness Model

Based on philosophical literature on PCE and on relevant work in moral theory, we have outlined two possible philosophical rationales for *Unacceptability*, that is the claim that PCE is morally unacceptable. According to the first rationale, the objection from fairness, *Unacceptability* can be rationalized by appeal to *Unfairness* and *Undeservingness*. According to the second rationale, the objection from hollowness, *Unacceptability* can be rationalized by appeal to *Hollowness* and *Undeservingness.*

We do not claim that these rationales constitute the only plausible ways of understanding the normative relationships between these judgments. For one thing, we have limited ourselves to rationales that can be understood as appealing to bad *consequences* of enhancement, yet we do not rule out the possibility that there are plausible motive- or means-based rationales for *Unacceptability*. For another, there may be consequence-based rationales for *Unacceptability* that we have not considered. We also do not claim that these rationales are in the end successful; indeed, one of us has previously argued against a view similar to *Undeservingness* (Douglas, 2014). However, we do claim the two rationales we have outlined are among the *prima facie* plausible rationales for *Unacceptability.*

Based on the idea that philosophical justifications can form the basis for psychological models, we derive two such models from our theoretical analyses above.

(1) **The Unfairness-Undeservingness Model**: People judge PCE to be unacceptable because they take it to produce unfairness and undermine the degree to which PCE-users deserve their achievement and associated reward. In other words, lay judgments of *Unacceptability* can be jointly explained by *Unfairness* and *Undeservingness*.
(2) **The Hollowness-Undeservingness Model**: People judge PCE to be unacceptable because they find achievements while using PCE hollow and undeserved. In other words, lay judgments of *Unacceptability* can be jointly explained by *Hollowness* and *Undeservingness*.

Note that in our philosophical analysis we discuss different possibilities for causal relationships between *Unfairness* and *Undeservingness* and between *Hollowness* and *Undeservingness*, respectively. For the sake of starting out with parsimonious models for empirical testing, we do not specify causal relationships beyond causes for *Unacceptability* in the psychological part. However, we return to the issue of a causal order of the explanatory variables in the discussion of our empirical results.

### Research Questions

The purpose of this paper is to combine normative philosophical and empirical psychological analyses to gain a deeper understanding of why people generally judge PCE to be morally unacceptable. We have derived two philosophically informed models for possible psychological explanations. Based on our theoretical analyses, we formulate the following two research questions.

(I) How well can the judgments *Undeservingness*, *Unfairness*, and *Hollowness* explain *Unacceptability*?
(II) How do these judgments interact, that is, more specifically: which of the two models, the Unfairness-Undeservingness Model or the Hollowness-Undeservingness Model, is better supported by empirical data?

In what follows, we report a test of these philosophy-grounded research questions against empirical data.

## Psychological Explanations

### Methods

We tested our research questions by re-analyzing parts of a larger data set we had collected and reported on previously (for details, see Faber et al., 2015a). For 94 participants, this data set contains information on the PCE-related judgments of interest, that is answers on *Undeservingness*, *Unfairness*, *Hollowness*, and *Unacceptability*. (The other participants in the complete data set did not answer questions in relation to cognitive enhancement but on motivation enhancement, so their judgments are not relevant for the present study. Please see Faber et al. (2015a) for further details on this data set.) Hence, our present sample contained 94 U.S. American participants (48% female, mean age 36.9 years^4^), who indicated that they had not previously used PCE. All respondents completed the study online. They gave informed consent to participate and were compensated financially for their participation. This study had been reviewed and approved by the University of Oxford’s Medical Sciences Interdivisional Research Ethics Committee.

After answering demographic questions, each participant read a hypothetical scenario about a male student who uses PCE. The part of the scenario describing this use read as follows: “While preparing for his exams, Alex takes medical substances to help him with his work. These pills normally are available on prescription only to treat certain diseases, but Alex knows that they improve brain performance in healthy people. They can make people think faster and more clearly. By taking these “smart pills,” he hopes to do better in his exams.” After participants had read the scenario, they answered several questions on 7-point Likert-scales (1 = “completely disagree”; 7 = “completely agree”). There was one item each for *Undeservingness* (“If Alex does well in his exams, he deserves praise,” reversely coded) and for *Hollowness* (“If Alex does well in his exams, it will be a hollow achievement”). To capture the frequent use of the more familiar concept of “cheating” to express concerns about unfairness, we included two items for *Unfairness*, one referring explicitly to the concept of unfairness (“It will be unfair if Alex does better in his exams than his classmates who don’t take the “smart pills”) and one to “cheating” (“Taking “smart pills” is cheating”). We used the mean of both items, which were highly correlated [*r*(92) = 0.842, *p* < 0.001], in subsequent analyses. (The pattern of results reported below remains unchanged when only the explicit unfairness item or the “cheating” item is included.) Finally, we assessed participants’ global judgment about *Unacceptability* (“Taking medical substances that improve smartness is acceptable”; reversely coded). (For further questions asked that are not relevant for this re-analysis and, hence, not reported below, see Faber et al., 2015a.)

### Results

To answer our research questions (I) how well the factors *Undeservingness*, *Unfairness*, and *Hollowness* can explain *Unacceptability*, and (II) which of the two proposed models, the Unfairness-Undeservingness Model and the Hollowness-Undeservingness Model, is better supported by our data, we used a regression-based approach.^5^

#### Descriptive Statistics

To begin with, to get a sense of the general view of *Unacceptability* in our sample, we performed a descriptive analysis. This analysis showed that the mean level of agreement that PCE is unacceptable was 4.70 (*SD* = 1.72); the median agreement was scale point 5 (“somewhat agree”). 58.6% of participants agreed (between strongly and somewhat) to *Unacceptability*, while 30.9% disagreed (between strongly and somewhat). The remaining 10.6% were undecided. Hence, in line with previous findings on non-users, participants in our sample on average exhibited support for *Unacceptability*, although there was a considerable variance in this view.

Similarly, we looked at the descriptive statistics for *Unfairness*, *Hollowness*, and *Undeservingness*. The mean level of agreement for *Unfairness* was 4.70 (*SD* = 1.76), and the median 5. The percentage of participants agreeing to *Unfairness* was 59.6%, and 27.7% disagreed. For *Hollowness*, the mean was 4.15 (*SD* = 1.79), and the median was 4. 45.7% of participants agreed to *Hollowness*, and 41.5% disagreed. For *Undeservingness*, the mean was 3.76 (*SD* = 1.61), the median 3. 33.1% agreed with *Undeservingness*, 51.1% disagreed. Hence, while the participants in our sample judged PCE as unfair on average, they were divided on the view whether its use makes achievements hollow, and overall did not agree with the claim that achievements gained with PCE are generally undeserved.

#### The Unfairness-Undeservingness Model

We tested the degree to which variations in agreement to *Unfairness* and *Undeservingness* could explain variations in agreement to *Unacceptability*, thereby evaluating the ability of the Unfairness-Undeservingness Model to explain the perceived unacceptability of PCE.

We conducted a linear regression analysis with *Unacceptability* as dependent variable and *Unfairness* and *Undeservingness* as predictors. Our two predictors explained a significant amount of the variance in the dependent variable [*F*(2,91) = 27.80, *p* < 0.001, *R^2^* = 0.379, *R^2^_adjusted_* = 0.366]. However, in this regression only *Unfairness* was a significant predictor of *Unacceptability* [β = 0.48, *t*(91) = 3.72, *p* < 0.001], while *Undeservingness* had no significant explanatory power beyond *Unacceptability* [β = 0.16, *t*(91) = 1.27, *p* = 0.208]. (*Unfairness* and *Undeservingness* were significantly correlated [*r*(92) = 0.769, *p* < 0.001], but multi-collinearity statistics showed no reason for concern in our data for this regression analysis (*Unfairness*: Tolerance = 0.409, VIF = 2.446; *Undeservingness*: Tolerance = 0.409, VIF = 2.446).

In sum, while the Unfairness-Undeservingness Model can account for about 38% of the variance in *Unacceptability* judgments, its explanatory power is mainly driven by *Unfairness*.

### The Hollowness-Undeservingness Model

Analogously to the calculations for the Unfairness-Undeservingness Model, we tested the plausibility of the Hollowness-Undeservingness Model in explaining *Unacceptability*.

A linear regression analysis with *Unacceptability* as dependent variable and *Hollowness* and *Undeservingness* as predictors showed that the two predictors significantly explained the dependent variable [*F*(2,91) = 22.72, *p* < 0.001, *R^2^* = 0.333, *R^2^_adjusted_* = 0.318]. In this regression, *Hollowness* was a significant predictor of *Unacceptability* [β = 0.35, *t*(91) = 2.57, *p* = 0.012], and *Undeservingness* had marginally significant explanatory power [β = 0.26, *t*(91) = 1.89, *p* = 0.062]. (*Hollowness* and *Undeservingness* were significantly correlated [*r*(92) = 0.781, *p* < 0.001], but multi-collinearity statistics showed no reason for concern regarding the reliability of our data (*Hollowness*: Tolerance = 0.390, VIF = 2.564*; Undeservingness*: Tolerance = 0.390, VIF = 2.564).

In sum, when regarded on its own (i.e., not in comparison to the Unfairness-Undeservingness Model), the Hollowness-Undeservingness Model explains about 33% of *Unacceptability*, with the influence of *Undeservingness* being only marginally significant.

#### Comparing the Unfairness-Undeservingness Model and the Hollowness-Undeservingness Model

In a further step, we compared the Hollowness-Undeservingness Model to the Unfairness-Undeservingness Model, looking at whether the former has any power in explaining *Unacceptability* beyond the Unfairness-Undeservingness Model.

We used all three factors *Unfairness, Hollowness*, and *Undeservingness*, as predictors in a linear regression with *Unacceptability* as dependent variable. We found that *Hollowness* as an additional predictor only added 1.2% to the explanatory power of the Unfairness-Undeservingness Model, which is a non-significant change [*F*(1,90) = 1.73, *p* = 0.193, *R^2^* = 0.391, *R^2^_adjusted_* = 0.371]. Correspondingly, with all three predictors in the regression analysis, only *Unfairness* had a significant influence on *Unacceptability* [β = 0.41, *t*(90) = 2.93, *p* = 0.004], while both *Undeservingness* [β = 0.07, *t*(90) = 0.50, *p* = 0.662] and *Hollowness* [β = 0.19, *t*(91) = 1.31, *p* = 0.193] had none. Again, *Hollowness* was significantly correlated with both *Undeservingness* [*r*(92) = 0.781, *p* < 0.001] and *Unfairness* [*r*(92) = 0.757, *p* < 0.001], but collinearity statistics seemed unproblematic (*Unfairness*: Tolerance = 0.346, VIF = 2.888; *Hollowness*: Tolerance = 0.330, VIF = 3.027*; Undeservingness*: Tolerance = 0.316, VIF = 3.164).

This model comparison reveals the importance of *Unfairness* in explaining *Unacceptability*. Both *Hollowness* [β = 0.55, *t*(92) = 6.38, *p* = 0.001] and *Undeservingness* [β = 0.53, *t*(92) = 6.05, *p* < 0.001] are significantly associated with *Unacceptability* when considered on their own, that is, as sole predictors. As soon as *Unfairness* is taken into account, however, they do not show any additional power in explaining *Unacceptability.* Put differently, while all three factors *Unfairness*, *Hollowness*, and *Undeservingness* jointly can explain about 39% of *Unacceptability*, *Unfairness* alone already explains about 36% [*F*(1,92) = 53.64, *p* < 0.001, *R^2^* = 0.361, *R^2^_adjusted_* = 0.361]. This 2.3% improvement in explanation *Hollowness* and *Undeservingness* can bring is statistically insignificant (*p* = 0.193, as reported above).

In sum, this analysis showed that the Unfairness-Undeservingness Model is superior to the Hollowness-Undeservingness Model in explaining *Unacceptability*, and that this superiority is driven by *Unfairness*. Amongst the three predictors *Unfairness*, *Hollowness*, and *Undeservingness*, *Unfairness* is the only one making a contribution in explaining *Unacceptability* beyond the two others.

## Discussion

In this paper, we aimed to gain a deeper understanding of why people generally endorse *Unacceptability*, that is judge PCE as morally unacceptable. For that, we combined normative philosophical and empirical psychological analyses.

### The Central Role of Unfairness in Explaining the Unacceptability of PCE

Based on philosophical literature, we argued that three judgments could be deployed to normatively rationalize *Unacceptability*, namely *Unfairness* (the idea that PCE produces unfair outcomes), *Hollowness* (the idea that achievements gained with PCE are hollow achievements), and *Undeservingness* (the idea that users of PCE are less deserving of reward). We developed philosophical rationales that combined these three judgments in different ways and, based on these rationales, proposed two psychological models that could potentially explain why lay people^4^ endorse *Unacceptability*. The Unfairness-Undeservingness Model holds that judgments of *Unacceptability* can be jointly explained by *Unfairness* and *Undeservingness*, and the Hollowness-Undeservingness Model holds that judgments of *Unacceptability* can be jointly explained by *Hollowness* and *Undeservingness*. We formulated two research questions: (I) How well can *Undeservingness*, *Unfairness*, and *Hollowness* can explain *Unacceptability*? And (II) is the Unfairness-Undeservingness Model or the Hollowness-Undeservingness Model better supported by empirical data?

We then tested these two research questions in a sample of lay people who indicated that they had not previously used PCE, using a regression-based approach. Descriptively, while participants tended to agree with the overall statements that PCE is unacceptable (*Unacceptability*) and with the claim that it is unfair (*Unfairness*), they were divided on the question whether it leads to achievements being hollow (*Hollowness*), and, on average, they tended to disagree with the idea that achievements gained with PCE are undeserved (*Undeservingness*).

With regards to our first research question, we found that *Unfairness* was clearly the strongest predictor of *Unacceptability*, explaining about 36% of the variance in *Unacceptability* judgments. While the two remaining judgments, *Hollowness* and *Undeservingness*, were also able to significantly predict *Unacceptability* when considered as sole predictors, they had no significant influence over and above *Unfairness*. All three predictors combined explained about 39% of variance. In other words, although people who judge PCE to be unacceptable also judge accomplishments gained with help of PCE to be undeserved and these achievements to be hollow, the two latter factors seem not to be necessary to explain why people endorse *Unacceptability.* All they can contribute to the explanation is just as well explained by *Unfairness* alone. Concerns about unfairness, on the other hand, seem to be central in understanding why PCE is judged as unacceptable.

With regards to our second research question, we consequently found that the Unfairness-Undeservingness Model was superior to the Hollowness-Undeservingness Model in explaining *Unacceptability*. While, again, the Hollowness-Undeservingness Model appeared to well explain *Unacceptability* when regarded on its own, a direct comparison to the Unfairness-Undeservingness Model showed that it did not make any contribution to understanding why PCE is judged as unacceptable beyond what we gain from the Unfairness-Undeservingness Model. Hence, if we are to accept one of these models, we should accept the Unfairness-Undeservingness Model.

Importantly, however, in the Undeservingness-Unfairness Model, *Unfairness* was the only predictor to make a significant contribution in explaining *Unacceptability*, while *Undeservingness* was not. What implications does this fact have for the Unfairness-Undeservingness Model?

### An “Unfairness Model” or *Unfairness* as Mediating Variable?

When we proposed the Unfairness-Undeservingness Model, we hypothesized that “people find PCE unacceptable because they take it to produce unfairness and undermine the degree to which the PCE-user deserves her achievement and associated reward. In other words, lay judgments of *Unacceptability* can be jointly explained by *Unfairness* and *Undeservingness*.” We found, however, that when we have knowledge about *Unfairness*, we do not need *Undeservingness* to explain *Unacceptability*. There seem to be two plausible possibilities of how this can be interpreted. It could be taken to support either a single-factor “Unfairness Model,” or the view that *Unfairness* acts as the mediating variable within the Unfairness-Undeservingness Model.

The straight-forward conclusion from our findings would be to propose a model we could call the “Unfairness Model.” An ideal model is one that offers a good trade-off between parsimoniousness and explanatory power. As *Unfairness* alone explains *Unacceptability* just as well as the Unfairness-Undeservingness Model, it seems appropriate to just reject *Undeservingness* and to propose a model that is based solely on *Unfairness*. This Unfairness Model could, of course, not fully explain why people judge PCE as morally unacceptable, but it could explain around 36% of variance in *Unacceptability* judgments, which is a considerable amount. Proposing such an Unfairness Model would imply that *Undeservingness* (and also *Hollowness*) are purely epiphenomenal. That is, people find PCE morally unacceptable because they find it unfair. And, when they find it unfair, then they judge achievements realized with it to also be undeserved (and hollow). This would be consistent with the view that *Unfairness* may rationalize *Undeservingness*, rather than the reverse (cf. section The Objection from Fairness).

There is, however, a second possibility that is consistent with our data. The Unfairness-Undeservingness Model could still be a plausible model, with the relationship between *Undeservingness* and *Unacceptability* being mediated by *Unfairness.* As described above, our original version of the Unfairness-Undeservingness Model proposed that “judgments of *Unacceptability* can be jointly explained by *Unfairness* and *Undeservingness*.” While it seems that “jointly” is not correct (as Undeservingness doesn’t add anything to this joint explanation), it might be that *Undeservingness* influences *Unacceptability* via *Unfairness*. This would imply that people find PCE unacceptable *because* they find it unfair, and they find it unfair *because* they find achievements realized with it undeserved. Such a causal chain would be in line both with our data and with philosophical considerations. While we find *Undeservingness* to be a significant predictor of *Unacceptability*, this relationship breaks down as soon as we add *Unfairness* as a second predictor. If, statistically, *Unfairness* were a full mediator of the relationship between *Undeservingness* and *Unacceptability*, we would expect such a result. Moreover, while no causal order between the variables *Unfairness* and *Undeservingness* has been assumed in our psychological model, it has been implicit in our philosophical rationales: in the section on “the objection from fairness,” we suggested that *Undeservingness* may rationalize *Unfairness* which in turn may rationalize *Unacceptability.* Hence, our philosophical analysis suggests a causal chain leading from *Undeservingness* over *Unfairness* to *Unacceptability*.

Unfortunately, based on our analyses we cannot assess which of the above possibilities (a single-factor Unfairness Model or *Unfairness* as the mediating variable in the Unfairness-Undeservingness Model) is true. Path analyses could give a good indication in larger samples, and controlled experiments could provide strong conclusions. We hope that future research will shed further light on the relationship between *Undeservingness* and *Unfairness*.

Importantly, however, both possibilities have at their core the same conclusion, namely that *Unfairness* plays a central role in explaining *Unacceptability*, and that we would need to understand why people find PCE unfair if we want to understand why they find it morally unacceptable. Or, put differently, it might well be that a lot of support for the view that PCE is unacceptable would dissolve if PCE was seen as fair. And indeed, concerns about the unfairness of PCE loom large in both the normative debate (e.g., Fukuyama, 2002; The President’s Council on Bioethics (U.S.), 2003; Gazzaniga, 2006; Rose, 2006) and lay people’s concerns (e.g., Forlini and Racine, 2012; Scheske and Schnall, 2012; Bossaer et al., 2013; Dubljevic et al., 2014; Santoni de Sio et al., in press for a review, see Schelle et al., 2014, p. 8–11). However, again, to date we cannot be certain what the *causal* relationship between *Unfairness* and *Unacceptability* is. So while PCE could be seen as unacceptable *because* it is seen as unfair, it might also be the other way around (PCE may be seen as unfair because seen as unacceptable), or bi-directional.

### Understanding the Psychology of PCE

The approach followed in this paper had two core elements. First, we took an interdisciplinary stance by combining normative philosophical and empirical psychological analyses. Second, we tried to shed light on how different normative judgments on PCE are related to each other psychologically. We hope that our approach has not only helped to advance research on the specific question why PCE is generally found unacceptable, but also to illustrate how philosophical analyses can be helpful in understanding the psychology of PCE.

With regards to interdisciplinarity, we hope to have shown how hypotheses derived from philosophical reasoning can serve as guideline about which psychological relationships are fruitful for testing. It would also be interesting, we think, to explore the reverse strategy, that is to use psychological findings to generate philosophical “hypotheses” than can be tested by normative or conceptual analyses. It might, for example, be worthwhile for philosophers to consider whether *Undeservingness* and *Hollowness* could be normatively epiphenomenal, in the sense that they are implications of *Unfairness* but play no role in the rationalization of *Unacceptability* by *Unfairness.*

With regards to our aim to test relations between different judgments on PCE, we think that this is not only worthwhile, but necessary both from an academic and a practical perspective. When we want to understand the psychology of cognitive enhancement, that is how human beings react to PCE and other mind-altering technologies, we need to gain more than a list of reactions these technologies evoke. Rather, we need to know which reactions are cause, and which are consequence; which are central and which are epiphenomenal.

Understanding the psychology of PCE, in turn, is necessary to estimate the non-pharmacological consequences of PCE use. Psychological reactions based on subjective judgments about PCE can be powerful. For instance, people tend to subjectively judge PCE as more effective than it actually is (Ilieva et al., 2013) and some employ it to cope with elevated stress (e.g., Wiegel et al., 2015). However, consuming PCE seems to be detrimental to reducing stress, but on the contrary weakens the protective effect of internal personal resources against burnout (Wolff et al., 2014). Moreover, it has been argued that the prevalent negative judgments of others regarding PCE can cause considerable psychological costs for users (for example reduced self-esteem; Faulmüller et al., 2013).

Increased understanding of psychological processes is also crucial for assessing the consequences PCE has beyond individual users. Current pharmacological research on the effectiveness of PCE substances measures how they influence participants’ individual performance. Based on such research, it has been argued that the use of PCE would also be beneficial on a societal level, for example, because enhancements will increase human productivity, resulting in general economic benefits through either greater availability of goods or lower prices (Bostrom and Ord, 2006; Buchanan, 2008, 2011a,b). However, a psychological understanding of normative attitudes to enhancement could complicate this picture. Employing a psychological perspective, it has been illustrated that the effect of PCE on an individual’s performance can be increased, but also be reduced, completely eliminated or even reversed at a group level (Faber et al., 2015b): The effectiveness of PCE in improving group performance depends on the psychological processes within the group, which, in turn, is guided by the subjective judgments the group members make about PCE. If, for example, group members who do not use PCE form negative attitudes to PCE-users, this can lead to these two parties not interacting efficiently and not functioning well as a performance group. In such a case, even though a PCE substance is an enhancement of individual performance (for pharmacological reasons), it could even act as an impairment for a group (for psychological reasons). Therefore, subjective judgments about PCE can determine the performance benefits groups can – or cannot – draw from PCE.

Hence, if we want to know how PCE affects us as a society, we need to understand not only the pharmacology, but also the psychology associated with such technologies. We think that both employing an interdisciplinary perspective and investigating the relationships between judgments on PCE is fruitful to understand this psychology. At present, research on the public perception of PCE and its consequences is still in its infancy. We hope that in the near future we will have a more comprehensive and coherent picture of the psychology of PCE – both for our academic understanding of human enhancement and to assist policy making.

## Author Contributions

NF and TD developed the models. NF analyzed the data. NF, JS, and TD wrote the paper.

## Conflict of Interest Statement

The authors declare that the research was conducted in the absence of any commercial or financial relationships that could be construed as a potential conflict of interest.

## Footnotes

1 Consequentialist moral theories hold that the moral status of an act is determined solely by its consequences (cf. Skorupski, 1995; Sinnott-Armstrong, 2001). On this view, the only reason that an act can be morally unacceptable (or “impermissible” or, simply, “wrong”) is that it has or can be expected to have bad consequences, or worse consequences than the alternatives. Deontological and virtue-ethical moral theories, such as those advanced by Immanuel Kant (e.g., 1786/2013) and Aristotle (e.g., trans, 2014) and their respective followers, hold that further considerations may be relevant. On most such theories, an act can be unacceptable even though it produces good (or the best possible) consequences. For instance, on a deontological theory, a good-maximizing act may be unacceptable because it violates someone’s rights or breaks the terms of a contract. On a virtue-ethical theory, it may be unacceptable because it is not what a virtuous agent would have done. However, deontological and virtue-ethical theories typically allow that the value of the consequences of an act remain relevant to the moral status of the act, and that an act can in some cases be morally unacceptable because it produces bad consequences which there is a duty not to produce, or which a fully virtuous agent would not produce (cf. Bennett, 1989; Hursthouse, 1991).

2 A third possibility is that *Unfairness* and *Undeservingness* are logically connected though neither is more fundamental than the other. For instance, it may be that to say that *X* enjoys an unfair advantage over *Y* is just to say that *A* does not deserve her advantage.

3 Again, however, other conceptions of the relationship between *Deservingness* and *Hollowness* are also plausible. For instance, it may be that when a person realizes an achievement without making the appropriate kind of human contribution, this independently renders both the achievement hollow and the achiever undeserving of reward.

4 Our participants were of mixed gender, age, as well as educational and socioeconomic backgrounds. They all lived in the USA, however, and in this sense our sample is quite restricted. We cannot be sure that our results can be generalized to people from other cultural backgrounds.

5 Using regression analyses seemed most appropriate to us given our specific research questions, but also in light of the ongoing debate on the statistical (in)appropriateness of dichotomizing continuous variables via median splits to use ANOVAs (e.g., Rucker et al., 2015).
